# Renal ADAM10 and 17: Their Physiological and Medical Meanings

**DOI:** 10.3389/fcell.2018.00153

**Published:** 2018-11-06

**Authors:** Takashi Kato, Man Hagiyama, Akihiko Ito

**Affiliations:** ^1^Department of Cell Biology, Johns Hopkins University School of Medicine, Baltimore, MD, United States; ^2^Department of Pathology, Kindai University School of Medicine, Osakasayama, Japan

**Keywords:** ADAM10 metalloprotease, ADAM17, kidney, ectodomain shedding, therapeutic inhibitors

## Abstract

A disintegrin and metalloproteinases (ADAMs) are a Zn^2+^-dependent transmembrane and secreted metalloprotease superfamily, so-called “molecular scissors,” and they consist of an N-terminal signal sequence, a prodomain, zinc-binding metalloprotease domain, disintegrin domain, cysteine-rich domain, transmembrane domain and cytoplasmic tail. ADAMs perform proteolytic processing of the ectodomains of diverse transmembrane molecules into bioactive mediators. This review summarizes on their most well-known members, ADAM10 and 17, focusing on the kidneys. ADAM10 is expressed in renal tubular cells and affects the expression of specific brush border genes, and its activation is involved in some renal diseases. ADAM17 is weakly expressed in normal kidneys, but its expression is markedly induced in the tubules, capillaries, glomeruli, and mesangium, and it is involved in interstitial fibrosis and tubular atrophy. So far, the various substrates have been identified in the kidneys. Shedding fragments become released ligands, such as Notch and EGFR ligands, and act as the chemoattractant factors including CXCL16. Their ectodomain shedding is closely correlated with pathological factors, which include inflammation, interstitial fibrosis, and renal injury. Also, the substrates of both ADAMs contain the molecules that play important roles at the plasma membrane, such as meaprin, E-cadherin, Klotho, and CADM1. By being released into urine, the shedding products could be useful for biomarkers of renal diseases, but ADAM10 and 17 *per se* are also notable as biomarkers. Furthermore, ADAM10 and/or 17 inhibitions based on various strategies such as small molecules, antibodies, and their recombinant prodomains are valuable, because they potentially protect renal tissues and promote renal regeneration. Although temporal and spatial regulations of inhibitors are problems to be solved, their inhibitors could be useful for renal diseases.

## ADAM10 and ADAM17

**FIGURE 1:**
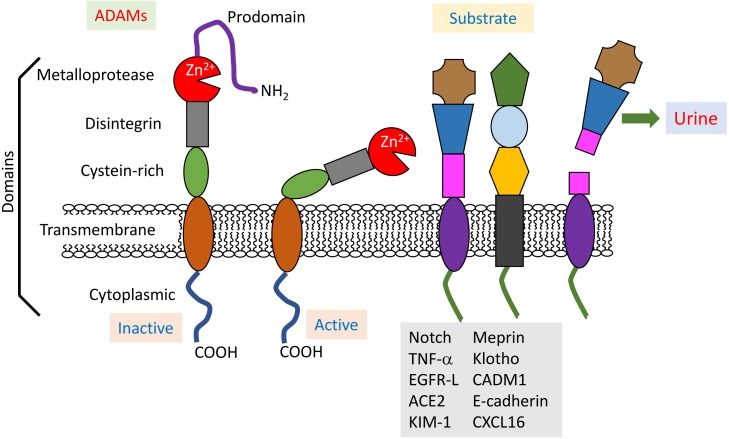
Structure and physiopathology of ADAMs. ADAMs display common domains (prodomain, metalloprotease, disintegrin, cysteine-rich, transmembrane, and cytoplasmic), and their activities are regulated by the prodomain. Active ADAMs cleave various membranous proteins as substrates in the kidneys. Some shedding fragments are detected in urine, and thus useful for the diagnosis of renal injuries.

A disintegrin and metalloproteinases (ADAMs), a superfamily of Zn^2+^-dependent transmembrane and secreted metalloproteases, are responsible for a large proportion of transmembrane protein cleavage. ADAMs are approximately 750 amino acids long and evolutionarily conserved, and 22 ADAM genes have already been identified in humans. ADAMs cleave a variety of transmembrane proteins at the plasma membrane, a process which is known as ectodomain shedding (Wetzel et al., 2017). ADAM10 and 17 consist of an N-terminal signal sequence, prodomain, metalloprotease (or catalytic) domain, disintegrin domain, cysteine-rich region, transmembrane region and cytoplasmic tail (Klein and Bischoff, 2011; Figure 1). In the catalytic active metalloprotease domain, a characteristic HExxHxxGxxH is commonly found (x: any amino acid residue) as a zinc-binding motif (Bode et al., 1993). Although these ADAMs are close relatives, their protein sequence homology is less than 30% (Gooz, 2010).

ADAM10 is ubiquitously expressed in various mammalian cells and reacts with more than 40 substrates (Dreymueller et al., 2012; Saftig and Lichtenthaler, 2015). ADAM10 is indispensable for embryonic development, because ADAM10 knockout (KO) mice die at embryonic day 9.5 and display a defective neuronal and vascular phenotype (Hartmann et al., 2002). With respect to the kidneys, ADAM10 is expressed in renal tubular cells, and its activity affects the expression of specific brush-border genes (Cong et al., 2011). Furthermore, ADAM10 has effects some renal diseases such as lupus nephritis, arterionephrosclerosis, and DN (Gutwein et al., 2009a; Hu et al., 2016; Lattenist et al., 2016; Orme et al., 2016).

ADAM17, also named TACE (TNF-α converting enzyme), is the most widely studied, and releases the ectodomain of various substrates from their transmembrane preforms to produce active soluble ligands. After ectodomain shedding, these ligands bind to receptors, which lead to downstream signaling. ADAM17 is widely expressed in various tissues including the kidney, and its expression changes during embryonic development and adult life (Black et al., 1997). Especially, ADAM17 is required for normal development, as its KO mice die during late development or soon after birth (Peschon et al., 1998). Since ADAM17 KO mice have a similar phenotype to EGFR KO mice, defects of the eyes, skin, heart, lungs, and hair (Miettinen et al., 1995), the precursor forms of the EGFR ligands are likely to be the main substrates of ADAM17. In addition, many reports suggest critical roles of ADAM17 in immunity, inflammation, and bone formation (Scheller et al., 2011; Dreymueller et al., 2012; Rose-John, 2013). ADAM17 is weakly expressed in proximal convoluted tubules (PCT), peritubular capillaries, glomerular endothelium, and podocytes in normal kidneys (Mulder et al., 2012; Perna et al., 2017). However, in the presence of interstitial fibrosis and tubular atrophy, ADAM17 expression is markedly upregulated in the tubules, capillaries, glomeruli, and in mesangium *de novo*.

## Substrates of ADAM10 and 17 in Kidneys (Summarized in Table 1)

### Cell Adhesion Molecule 1 (CADM1)

Cell adhesion molecule 1 (CADM1) is an intercellular adhesion molecule that belongs to the immunoglobulin (Ig) superfamily, and it is localized on the lateral cell membrane and mediates neighboring cell–cell binding (Murakami, 2005; Ito et al., 2012). It functions by transmitting cell attachment signals to promote actin reorganization in the cytoplasm (Kato et al., 2018). Various types of epithelial cells express CADM1, including pulmonary cells and renal distal tubules (Nagata et al., 2012; Kato et al., 2018). CADM1 is cleaved at its ectodomain, yielding a C-terminal fragment, αCTF (Mimae et al., 2014). ADAM10-dependent CADM1 shedding occurs in emphysematous lungs, and αCTF contributes to apoptosis of lung epithelial cells (Nagara et al., 2012; Mimae et al., 2014). Similarly, CADM1 α-shedding and αCTF enhancement were found in human nephropathies, such as arterionephrosclerosis (AS) and diabetic nephropathy (DN) (Kato et al., 2018). In particular, reduction of the full-length CADM1 (FL-CADM1) level was correlated with tubular epithelial cell (TEC) apoptosis and increases of blood urea nitrogen (BUN) and serum creatinine (sCre). By conducting the *in vitro* studies, it may be found that CADM1 ectodomain shedding could contribute to the development of chronic kidney disease (CKD).

**Table 1:** Substrates for ADAM 10 and 17 in the kidneys.

Substrates	ADAMs	Associated diseases
CADM1	10	Diabetic nephropathy, arterionephroscrelosis
E-cadherin	10	Autosomal dominant polycystic kidney disease
CXCL16	10	Lupus nephritis, acute tubular necrosis
TNF-α	17	Lupus nephritis, diabetic nephropathy, acute kidney injury
EGFR ligands	17	Renal fibrosis, polycystic kidney disease
ACE2	17	Diabetic nephropathy
KIM-1	17	Acute kidney injury
Notch	10, 17	Renal fibrosis, glomerulosclerosis, diabetic nephropathy
Meprin	10, 17	Acute kidney injury
Klotho	10, 17	Hyperphosphatemia


### E-cadherin

E-cadherin forms adherens junctions between areas of cell–cell contact through its ectodomain, and it plays crucial roles in the integrity of cellular polarity and cell–cell adhesions (Gall and Frampton, 2013). It can be removed from the cell surface by proteolytic cleavage as soluble E-cadherin (sE-cad), which has been reported in patients with organ failure. ADAM10 is one of several proteases that cleave E-cadherin (Crawford et al., 2009; Ma et al., 2016). The increased shedding of E-cadherin was blocked by ADAM10 inhibition (Xu et al., 2015). The effects of ADAM10 activation on E-cadherin shedding was actually reported in ADPKD (autosomal dominant polycystic kidney disease). *Pkd1* (an ADPKD responsible gene) mutation or deletion promotes the maturation of ADAM10 via Gα12 activation, which increases E-cadherin shedding and results in the cystogenesis of renal TECs.

### CXCL16

CXCL16 not only functions as an adhesion molecule for CXCR6, but also plays an important role as a scavenger receptor for oxidized low-density lipoprotein (oxLDL) (Minami et al., 2001; Shimaoka et al., 2004; Gutwein et al., 2009b). The human kidneys highly express CXCL16 mainly in the distal convoluted tubule (DCT), connecting tubule (CNT), and collecting duct, and CXCL16 and ADAM10 are also expressed in podocytes (Gutwein et al., 2009b). Elevated CXCL16 cleavage was accompanied by increased levels of oxLDL in an atherosclerosis and CKD model (Okamura et al., 2007). ADAM10 and 17 are mainly involved in CXCL16 release from the cell membrane (Abel et al., 2004; Gough et al., 2004). Thus, both ADAMs promoted the accumulation of oxLDL, which activates proinflammatory pathways, and then causes collagen synthesis and fibrosis. The increase of urinary CXCL16 has been detected in patients with acute tubular necrosis or with lupus nephritis (Wu et al., 2007; Schramme et al., 2008), revealing that CXCL16 could be a useful biomarker for these diseases. A soluble form of CXCL16, proteolytically released, acts as a chemotactic factor. Renal allograft biopsies with acute interstitial rejection showed increased ADAM10 expression. Thus, CXCL16 and ADAM10 are involved in the recruitment of T cells to the kidney and play a substantive role in inflammatory renal diseases (Schramme et al., 2008).

### Tumor Necrosis Factor (TNF)-α

Proinflammatory tumor necrosis factor (TNF)-α belongs to a family of both soluble and cell-bound cytokines, and it is produced by immune cells and vascular endothelial cells, but also renal TECs and mesangial cells (Mehaffey and Majid, 2017). TNF-α and its receptors may be related to kidney injury (Ernandez and Mayadas, 2009). The involvement of TNF-α in renal injuries has been suggested in the presence of various renal injuries, such as lupus nephritis, DN, acute kidney injury (AKI), cisplatin-induced renal injury, renal ischemia/reperfusion injury, and kidney allograft rejection (Sanchez-Niño et al., 2010). TNF-α activation is closely correlated with ADAM17’s activity in the kidney. Actually, TNF-α cleavage and release were significantly downregulated in proximal TEC-specific conditional ADAM17 KO mice, and they exhibited markedly suppression in renal proinflammatory markers and the infiltration of macrophages and neutrophils following renal injury (Kefaloyianni et al., 2016).

### Epidermal Growth Factor Receptor (EGFR) Ligands

Two epidermal growth factor receptor (EGFR) ligands, heparin-binding (HB)-EGF and transforming growth factor (TGF)-α, are involved in proliferative, migratory, and fibrotic responses of tubular cells. Elevated ADAM17 activity causes sustained EGFR activation and fibrosis after kidney injury (Kefaloyianni et al., 2016). The increased EGFR signaling through TGF-α or HB-EGF was shown in several renal diseases including polycystic kidney disease (PKD) (Richards et al., 1998). In a model mouse of autosomal recessive PKD, increased TGF-α expression was noted in the PCTs of cystic kidneys (Dell et al., 2001). Actually, an ADAM-17 inhibitor could significantly decrease cyst formation and improve the renal function (Nemo et al., 2005). Increased ADAM17 activity in the cystic kidneys, especially the collecting duct epithelial cells, leads to constitutive shedding of several growth factors, including HB-EGF and TGF-α. Their shedding maintains a higher cell proliferation rate in PKD cells. PKD cells then display increased lactate formation and extracellular acidification, indicative of aerobic glycolysis (Gooz et al., 2014).

### Angiotensin-Converting Enzyme 2 (ACE2)

Angiotensin-converting enzyme 2, highly expressed in renal PCTs, degrades the vasoconstrictor angiotensin II (ANG II) to ANG-(1-7) (Chodavarapu et al., 2013). It is shed from renal tubular cells into the urinary space, and two enzymatically active glycosylated fragments may be enhanced via ADAM17 activation in diabetes (Xiao et al., 2014). This shedding is stimulated by high glucose and Ang II, can increase Ang II-degrading products in the urine of DN patients, and could serve as a biomarker of early kidney injury (Xiao et al., 2014). Furthermore, urinary ADAM17 and its substrate, ACE2, are increased in diabetic patients and its model mice (Chodavarapu et al., 2013; Gutta et al., 2018), and the shedding fragments could also be an early biomarker to predict DN-induced CKD.

### Kidney Injury Molecule-1 (KIM-1)

Kidney injury molecule-1 is a receptor for phosphatidylserine, an efferocytosis signal on the surface of apoptotic cells that labels them for phagocytic clearance. Its expression is induced on PTECs in ischemic AKI, and KIM-1 ectodomain shedding generates a soluble fragment that serves as an important biomarker for AKI. Oxidative stress accelerated KIM-1 shedding (Gandhi et al., 2014). Of note, ADAM17 mediated this shedding of KIM-1 during injuries, and accelerated shedding inhibits efferocytosis (Gandhi et al., 2014).

### Notch

Notch is a critical regulator of renal development, and its signaling is involved in both acute and chronic kidney injuries (Sweetwyne et al., 2014). Its overexpression is causally associated with fibrosis in diverse organs and tissues, especially tubulointerstitial fibrosis and glomerulosclerosis (Sweetwyne et al., 2014). Notch functions via its ligand-receptor binding, but also as ectodomain shedding fragments by ADAM10 and 17 (Brou et al., 2000; Hu and Phan, 2016). This ectodomain shedding product is further cleaved by a γ-secretase complex, and released as the intracellular domain of Notch (NICD) (Fortini, 2002; Okochi et al., 2002). NICD translocates into the nucleus and then it modifies target gene expression, mainly Hes family members, which correlates with transforming growth factor-β-mediated epithelial-mesenchymal transition (Artavanis-Tsakonas et al., 1999; Zavadil et al., 2004). Both ADAMs thus play essential roles in Notch signal activation and renal fibrosis.

### Meprin

Meprins are also Zn^2+^-dependent metalloproteinases that are highly expressed at the brush-border membranes of the kidney and evolutionarily related to other proteases, MMPs and ADAMs (Stöcker et al., 1995), but possess unique structural and functional properties (Broder and Becker-Pauly, 2013). They can degrade numerous substrates such as basement membrane proteins (collagen, laminin, and fibronectin) and pro-cytokines, growth factors, and protein kinases (Herzog et al., 2014). Meprin A, composed of α and β subunits, is anchored to the plasma membranes via the transmembrane domain of the β subunits, and is the major form in the apical membranes of renal PCTs (Kumar and Bond, 2001; Bond et al., 2005; Sterchi et al., 2008). In IR-induced AKI, meprin β was shed from PCT membranes, and excreted into the urine. Thus, released meprin β may become detrimental during renal injury for its protein degradation activities. The meprin inhibitor actinonin exhibited strong protection against renal IR injury and hypoxia-reoxygenation injury (Carmago et al., 2002). Actinonin protected the renal morphology and lowered BUN and sCre levels in the presence of renal sepsis (Wang et al., 2011). ADAM10 is responsible for meprin β shedding, and thus the prevention of ADAM 10 activity could be of therapeutic benefit in AKI (Herzog et al., 2014). Also, a soluble form of meprin β is produced and released into urine after IR injury, and thus meprin β shedding also marked potential as a urine biomarker for renal injuries.

### Klotho

Klotho is known as an anti-aging protein, and its KO mice exhibit many changes during aging including atherosclerosis and have a short lifespan (Kuro-o et al., 1997). Membrane-bound klotho is predominantly expressed in the DCT and CNT (Kuro-o et al., 1997; Li et al., 2004). The gene for mammalian KL has two transcripts that encode a long type I transmembrane protein and a short secreted-protein. The extracellular domain of long-isoform KL is cleaved and released from the cell membrane (Matsumura et al., 1998). A key function of membrane-bound klotho is to act as an obligate cofactor for the fibroblast growth factor (FGF) receptor, thereby enhancing FGF23 signaling, and leading to enhanced phosphate excretion (Kurosu et al., 2006; Urakawa et al., 2006; Gattineni et al., 2009). ADAM10 is one candidate molecule for cleaving KL from the plasma membrane (Chen et al., 2007). Therefore, ADAM10 activation leads to the dysfunction of phosphate excretion (hyperphosphatemia).

## ADAM10 and ADAM17 as Clinical Targets

**FIGURE 2:**
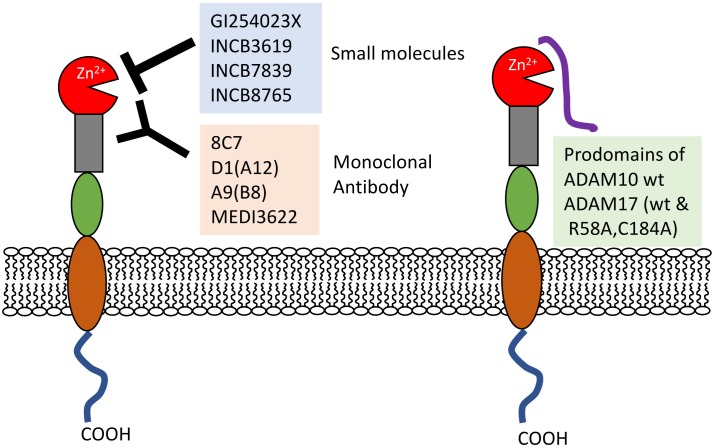
Inhibitors of ADAM10 and prodomains 17. Small molecule inhibitors and monoclonal antibodies directly prevent the interactions between proteases and substrates. Recombinant ADAM10 and 17 prodomain (wt and its mutant) close active sites. wt, wild-type.

ADAM10 and 17 are closely correlated with renal injuries including excess inflammation and tubular cell destruction. In addition to their substrates, ADAM10 and 17 *per se* are also important biomarkers of renal dysfunctions, such as early DN (Petrica et al., 2017; Gutta et al., 2018). Furthermore, many efforts have been made to develop strategies to block ADAM10 and 17 activities involving small molecules and monoclonal antibodies (Figure 2).

### Small Molecules (Hydroxamate-Based Compounds)

Many small-molecule ADAMs inhibitors have been developed and mainly tested in experimental cancer models. ADAM10 inhibitors could exhibit potency to prevent renal injury. GI254023X is a hydroxymate-based inhibitor, which has inhibitory potential by chelating Zn^2+^ of the active sites of protease (Dreymueller et al., 2015). GI254023X prevents ADAM10 activity more effectively than ADAM17 (Hundhausen et al., 2003; Ludwig et al., 2005). The advanced molecules INCB3619, INCB7839, and INCB8765 showed improved selectivity and bioavailability (Zhou et al., 2006; Fridman et al., 2007; Duffy et al., 2011; Mathews et al., 2011; Grabowska et al., 2012). The hydroxamate-based INCB3619 and INCB7839 inhibitors have dual effects on ADAM10 and 17 with high potency. As an ADAM17-selective inhibitor, KP457 inhibits ADAM17 with a much higher potency than ADAM10 and MMPs (Hirata et al., 2017). However, many hydroxamate-based compounds show hepatotoxicity, and so their clinical application requires close attention.

### Antibodies

Monoclonal antibodies (mAb) can overcome the problems of hydroxamate-based compounds. The 8C7 mAb masked the ADAM10 recognition pocket and was more efficient than GM6001, a broad-spectrum metalloprotease inhibitor (Atapattu et al., 2012). Because of ADAM10 suppression, the 8C7 antibody could inhibit tumor growth in mouse models, particularly regrowth after chemotherapy. Similarly, targeted inhibition of active ADAM10 might be a potential therapy for some kinds of renal injuries.

Also, ADAM17 antibodies were developed. D1(A12) antibody binds to both catalytic and non-catalytic domains of ADAM17 (Tape et al., 2011). However, D1(A12) does not react with murine ADAM17, because therapeutic strategies could not be developed in experimental animal models. Thus, the antibody A9(B8) recognizes both human and murine ADAM17 and is more efficient than D1(A12) (Kwok et al., 2014). The A9(B8) antibody was investigated in a mouse model of cardiac hypertrophy by AngII infusion (Takayanagi et al., 2016). This antibody did not affect AngII-induced hypertension, but prevented endoplasmic reticulum stress and cardiovascular remodeling, showing that ADAM17 inhibitors could be beneficial for the treatment of certain hypertensive conditions. MEDI3622, another antibody for ADAM17, was produced to bind to a unique hairpin loop in the ADAM17 structure, and it was useful in an EGFR-dependent tumor model (Rios-Doria et al., 2015; Peng et al., 2016; Dosch et al., 2017).

### Prodomain

The recombinant mouse ADAM10 prodomain is a potent competitive inhibitor of human ADAM10 activity with higher selectively (Moss et al., 2007).

ADAM17 prodomains could also be valuable inhibitors. A stable form of the auto-inhibitory TPD (TACE prodomain) inhibits ADAM17, but does not prevent the related ADAM10 activity (Wong et al., 2016). Furthermore, to create a more practical protein of TPD, Wong et al. produced a cleavage-resistant version (R58A) and disulfide-bond lost version (C184A) of the ADAM17 prodomain, and finally created the double mutant TPD (R58A and C184A). This mutant prodomain effectively modulated TNF-α secretion. TPD attenuated TACE-mediated disease models of sepsis, rheumatoid arthritis (RA), and inflammatory bowel disease (IBD) (Wong et al., 2016).

### Others

Some natural compounds reduce ADAM10 activity. Rapamycin suppresses ADAM10 activity (Zhang et al., 2010) and prevents organ rejection following transplantation via suppressive effects on ADAM10 activity. Fish oil (FO) supplement reduces the shedding and release of transmembrane proteins from endothelial cells by ADAM10 and 17, and thus prevents atherogenic diseases (Speck et al., 2015). By suppressing ADAM activity, FO partly contributes to an improved endothelial barrier function and prevents lipoprotein and macrophage accumulation, although the detailed mechanisms remain unknown. Furthermore, the diterpenoid epoxide triptolide downregulates ADAM10 expression, possibly through its degradation (Soundararajan et al., 2009). In traditional Chinese medicine, triptolide has been used for centuries to treat inflammatory diseases such as RA, systemic lupus erythematosus (SLE), and ADPKD (Leuenroth et al., 2007; Wetzel et al., 2017).

Because targeted inhibition of active ADAM10 and/or 17 is expected to become a potential therapy for associated diseases, these strategies have been advanced. However, ADAM10 and 17 have many substrates with diverse functions; therefore, it is important for the temporal and spatial regulation of inhibitors to avoid undesirable side effects.

## Author Contributions

TK conceived the idea and wrote the manuscript. MH and AI edited the manuscript and helped to improve the quality of this review paper.

## Conflict of Interest Statement

The authors declare that the research was conducted in the absence of any commercial or financial relationships that could be construed as a potential conflict of interest.
